# The Coupled Representation of Hierarchical Features for Mild Cognitive Impairment and Alzheimer's Disease Classification

**DOI:** 10.3389/fnins.2022.902528

**Published:** 2022-06-03

**Authors:** Ke Liu, Qing Li, Li Yao, Xiaojuan Guo

**Affiliations:** ^1^School of Artificial Intelligence, Beijing Normal University, Beijing, China; ^2^Engineering Research Center of Intelligent Technology and Educational Application, Beijing Normal University, Beijing, China; ^3^State Key Laboratory of Cognitive Neuroscience and Learning, Beijing Normal University, Beijing, China; ^4^Beijing Key Laboratory of Brain Imaging and Connectomics, Beijing Normal University, Beijing, China

**Keywords:** coupled interaction representation, hierarchical features, classification, mild cognitive impairment, Alzheimer's disease, structural MRI

## Abstract

Structural magnetic resonance imaging (MRI) features have played an increasingly crucial role in discriminating patients with Alzheimer's disease (AD) and mild cognitive impairment (MCI) from normal controls (NC). However, the large number of structural MRI studies only extracted low-level neuroimaging features or simply concatenated multitudinous features while ignoring the interregional covariate information. The appropriate representation and integration of multilevel features will be preferable for the precise discrimination in the progression of AD. In this study, we proposed a novel inter-coupled feature representation method and built an integration model considering the two-level (the regions of interest (ROI) level and the network level) coupled features based on structural MRI data. For the intra-coupled interactions about the network-level features, we performed the ROI-level (intra- and inter-) coupled interaction within each network by feature expansion and coupling learning. For the inter-coupled interaction of the network-level features, we measured the coupled relationships among different networks *via* Canonical correlation analysis. We evaluated the classification performance using coupled feature representations on the Alzheimer's Disease Neuroimaging Initiative (ADNI) database. Results showed that the coupled integration model with hierarchical features achieved the optimal classification performance with an accuracy of 90.44% for AD and NC groups, with an accuracy of 87.72% for the MCI converter (MCI-c) and MCI non-converter (MCI-nc) groups. These findings suggested that our two-level coupled interaction representation of hierarchical features has been the effective means for the precise discrimination of MCI-c from MCI-nc groups and, therefore, helpful in the characterization of different AD courses.

## Introduction

Alzheimer's disease (AD) is one of the most severe neurodegenerative dementias in the elderly, and mild cognitive impairment (MCI) is a prodromal stage with a higher risk of progression to AD in patients with MCI than normal controls (NC) (Dona et al., 2016; Arbabshirani et al., 2017; Zhang et al., 2021a). Neuroimaging techniques provide objective and effective tools to study the human brain and have been widely used in the diagnosis of AD or MCI from NC. Neuroimaging studies have shown remarkable structural and functional alterations in the human brain during the course of AD (Vemuri and Jack, 2010; Rathore et al., 2017; Leandrou et al., 2018). Structural magnetic resonance imaging (MRI) studies have extracted hierarchical features [the voxel level, the regions of interest (ROI) level, or the network level] as explicit variables to discriminate AD and MCI from NC. However, a large number of structural MRI studies only extracted low-level features, or simply concatenated multitudinous features, while ignoring the interregional covariate information among features (Anstey and Maller, 2003; Yang et al., 2011; Moradi et al., 2015; Hu et al., 2016; Rathore et al., 2017; Rondina et al., 2018), so they cannot fully exploit the latent and complex information integrated with hierarchical features. The effective feature representations help to enhance the performance of classification. Therefore, the appropriate representation and integration of multilevel features will be preferable for the precise discrimination of AD, MCI, and NC.

Based on structural MRI data, researchers extracted hierarchical imaging features, such as the gray matter (GM) density as the voxel-level features (Moradi et al., 2015; Zeifman et al., 2015), the average gray matter volume (GMV) of brain regions as the ROI-level features (Shi et al., 2014; Hu et al., 2016), or the independent components (ICs) from independent component analysis (ICA) as brain network-level features (Yang et al., 2011). Moradi et al. considered the smoothed GM density from structural MRI data as voxel-level features for AD conversion prediction in subjects with MCI (Moradi et al., 2015). In comparison with the voxel-level features with redundant information but expensive computation, ROI-level features significantly reduce the dimensionality of brain imaging data by uniting the structural adjacent voxels. The GMV from different ROIs has been applied as an independent variable to investigate the predictive power for distinguishing AD with MCI (Zhang et al., 2011) and classifying AD from NC (Rondina et al., 2018). ICA is a data-driven approach that decomposes the whole-brain voxel-vise information into a few maximally independent components based on inter-regional covariance relationships. The brain GM networks obtained from ICA have been considered as brain network-level features to differentiate individuals with AD and NCs, thus providing new avenues for the network-level features in AD classification (Yang et al., 2011; Wei et al., 2016). However, it has been noted that ROI-level features in the same network exhibited more complicated regional dependencies than those in different brain networks (Liu et al., 2017a; Rathore et al., 2017; Filippi et al., 2020; Feng et al., 2021). Nevertheless, the aforementioned studies mostly constructed classification models using the single level of features separately while neglecting the complex interaction relationships among multilevel features.

There were explicit and hidden coupled interactions, much more abundant than simple linear correlation among attributes or features of objects in many domains, like the recommender systems (Wang and Cao, 2020; Zhang et al., 2021b), outlier detection (Pang et al., 2016), and pieces of neuroscience research (Shi et al., 2014, 2015, 2020). Many coupled analysis models were proposed to analyze the explicit and hidden couplings and revealed the non-independent and identical distribution (non-IIDness) characteristics for different data types (Wang et al., 2013, 2015a,b). For numerical data, Wang et al. detailed the intra-coupled interaction to capture the correlations between a feature and its own expanded powers and the inter-coupled interaction to quantify the interactive relationships among each feature and the expanded powers of the other features (Wang et al., 2013). A few imaging studies investigated AD classification with the coupling characteristics of the ROI-level features (Shi et al., 2014, 2020). Although such studies demonstrated high accuracy for AD, MCI, and NC classifications with coupled feature analysis, they still weakened or overlooked the coupled relationship at network-level features. The ROI-level features within the same network strongly interacted with each other (Brickman et al., 2007). Different brain networks collaborated with each other and carried explicit or implicit relationships (Betzel et al., 2014; Zuo et al., 2017). Consequently, greater effort should be focused on designing an appropriate coupled interaction model to integrate the ROI-level and network-level coupling relationships.

To integrate the intrinsic coupling relationships of the ROI-level and network-level features from structural MRI data, we proposed a novel inter-coupled feature representation method for the network-level features and built a two-level (the ROI level and the network level) coupled feature integration model for AD, MCI, and NC classification. For the intra-coupled interactions about the network-level features, we performed the ROI-level (intra- and inter-) coupled interaction within each network by feature expansion and coupling learning. For the inter-coupled interaction of the network-level features, we introduced the measurement of the coupled relationships among different networks *via* Canonical correlation analysis (CCA). We compared the identification performances in AD, MCI, and NC classification with different feature representation models. We hypothesized that two-level (the ROI level and the network level) coupled feature integration models would achieve better or comparable AD classification performance.

## Materials and Methods

### Participants

This study included 121 patients with AD and 120 NC subjects, and 126 MCI converters (MCI-c) and 108 MCI non-converters (MCI-nc), with baseline structural MRI data from the Alzheimer's Disease Neuroimaging Initiative (ADNI) database (adni.loni.usc.edu). The up-to-date information on ADNI's general inclusion criteria is described at www.adni-info.org. Briefly, the subjects were between 55 and 90 years of age. General group inclusion/exclusion criteria were as follows: (1) NC subjects: Mini-Mental State Examination (MMSE) scores between 26 and 30, a Clinical Dementia Rating (CDR) score of 0, non-depressed, non-MCI, and non-demented; (2) AD subjects: MMSE scores <26, a CDR score of 0.5 or 1, and met the National Institute of Neurological and Communicative Disorders and Stroke and the Alzheimer's Disease and Related Disorders Association (NINCDS/ADRDA) criteria for probable AD diagnosis; and (3) MCI subjects who had a CDR score of 0.5, MMSE scores between 21 and 30, and memory complaints and abnormal memory function according to the Logical Memory II subscale (Delayed Paragraph Recall but an absence of dementia. The patients with MCI who converted to AD within 3-year follow-up were classified into the MCI-c group; otherwise, they were classified into the MCI-nc group. With respect to the gender ratio and age, the AD group did not significantly differ from the NC group (*p* = 0.14 in the gender ratio and *p* = 0.68 in age), and the MCI-c did not significantly differ from the MCI-nc group (*p* = 0.66 in the gender ratio and *p* = 0.20 in age). However, the AD group exhibited significantly lower MMSE scores (*p* = 1.25 *E* − 42) than the NC group. Table 1 lists the demographics of all these subjects.

**Table 1:** The characteristics of participants with AD, NC, MCI-c, and MCI-nc.

	**AD (*n* = 121)**	**NC (*n* = 120)**	**MCI-c (*n* = 126)**	**MCI-nc (*n* = 108)**
Age (years)	74.87 ± 8.07	75.26 ± 6.52	73.47 ± 7.23	73.33 ± 7.73
Gender (M/F)	70/51	58/62	77/49	69/39
Education (years)	15.72 ± 2.61	16.43 ± 2.74	16.09 ± 2.64	15.89 ± 2.63
MMSE score	21.71 ± 3.94	29.18 ± 0.98	26.88 ± 1.76	28.06 ± 1.75
APOE ε4 (NC/HT/HM)	41/80/0	79/33/8	37/65/24	67/35/6
ADAS-cog score	21.52 ± 7.96	5.76 ± 3.02	13.60 ± 4.64	8.03 ± 3.47
Conversion time (years)	–	–	1.48 ± 0.69	–

*AD, Alzheimer's disease; NC, normal control; MCI, mild cognitive impairment; MCI-c, MCI converter; MCI-nc, MCI non-converter; M/F, male/female; MMSE, Mini-Mental State Examination; APOE, apolipoprotein E; NC, non-carrier; HT, heterozygote; HM, homozygote; ADAS-cog, Alzheimer's Disease Assessment Scale-Cognitive Subscale*.

### Structural MRI Data Acquisition

Structural MRI images were acquired from multiple sites and platforms with different acquisition parameters, which can be found at http://adni.loni.usc.edu/methods/documents/mriprotocols/. The T1-weighted magnetization prepared rapid gradient echo (MPRAGE) images of all these subjects were obtained from 1.5T or 3T scanners. For intensity non-uniformity and gradient nonlinearity correction, the grad warp, B1 calibration, and N3 correction were implemented on each structural MRI image. The processed NIFTI images were downloaded for this study. Details of the protocols of MRI image correction can be found at http://adni.loni.usc.edu/methods/mri-analysis/mri-pre-processing/.

### Image Preprocessing

All of the spatial preprocessing of structural MRI images was performed *via* Statistical Parametric Mapping (SPM8) software (https://www.fil.ion.ucl.ac.uk/spm/software/spm8/) in MATLAB. The Voxel-Based Morphometry (VBM) Toolbox (http://dbm.neuro.uni-jena.de/wordpress/vbm/download/) was used for the automated segmentation and normalization of structural MRI images. First, each image was segmented into three parts: GM, white matter, and cerebrospinal fluid (CSF) (Rajapakse et al., 1997; Manjón et al., 2010). A de-noising filter and a classical Markov random field (MRF) approach were implemented to further improve the segmentation effect (Ashburner, 2007). Then, GM images were normalized by the Diffeomorphic Anatomical Registration using Exponential Lie Algebra (DARTEL) protocol and transformed into the Montreal Neurological Institute (MNI) space (Ashburner, 2007). Finally, all the subjects' GM images were smoothed with a kernel of 8-mm full width at half maximum (FWHM).

### Feature Extraction

In this study, the brain network-level features were extracted *via* ICA using the Fusion ICA toolbox (FIT) (https://trendscenter.org/software/fit/). The GM images of the AD and NC groups were decomposed into a mixing coefficient matrix and a source matrix with the Minimum Description Length (MDL) criteria to estimate the optimal number of ICs. Each row of the source matrix represents an IC, and each column of the mixing coefficient matrix represents the contribution of each subject to the corresponding IC. A two-sample *t*-test was performed on the mixing coefficient of each IC, and then these IC maps with significant between-group differences were converted to a z-score brain map and reshaped to a binarization mask with a threshold *Z* ≥ 3. For each IC, the main brain clusters were reported based on the Anatomical Automatic Labeling (AAL) atlas. For each subject in the AD, MCI-c, MCI-nc, and NC groups, only the top 3 ROIs ranked by the cluster size were selected as the ROI-level features within each network. The average GMV of each ROI falling into the brain network template was regarded as the ROI-level original feature value. The average GMV of voxels within each binarization network template was calculated as the value of network-level original features.

### Two-Level Coupled Feature Representation

We took the AD and NC groups as an example to illustrate the implementation of coupled feature representation at the ROI level and the network level. The two-level coupled feature representations of the MCI-c and MCI-nc groups were generated using the same method as the AD and NC groups.

Suppose that there are *m*_1_ samples in the AD group and *m*_2_ samples in the NC group (*M* = *m*_1_ + *m*_2_), we assume that there are *N* ROI-level original features and *L* brain network-level original features for each subject. For the *l*_*th*_ brain network, if there are *n* ROI-level original features (*n* × *L* = *N*), and the numerical value of the *k*_*th*_ ROI-level features of the *i*_*th*_ subject is denoted as ${\;z}_{i,k}^{\left(l\right)}$, then the ROI-level original feature vector can be represented as ${\text{z}}_{i}^{\left(l\right)}\in {\mathbb{R}}^{n}=\left[z_{i,1}^{\left(l\right)},z_{i,2}^{\left(l\right)},\cdots \;,z_{i,n}^{\left(l\right)}\right]$. The whole ROI-level original feature vector for the *i*_*th*_ subject is ${\text{z}}_{i}\in {\mathbb{R}}^{N}=\left[{\text{z}}_{i}^{\left(1\right)},{\text{z}}_{i}^{\left(2\right)},\cdots \;,{\text{z}}_{i}^{\left(L\right)}\right]$. For the *i*_*th*_ subject, the brain network-level original feature vector is ${\text{v}}_{i}\in {\mathbb{R}}^{L}=\left[v_{i,1},v_{i,2},\cdots \;,v_{i,L}\right]$, and the numerical value of the *j*_*th*_ network-level features is denoted as *v*_*i,j*_. The superscript ⊤ represents a transpose operator of a vector or a matrix. In particular, we considered two levels of feature representation with the **O**riginal **F**eatures **M**atrix (OFM): ${\text{Z}}_{\text{OFM}}={\left[{\text{z}}_{1},\cdots \;,{\;\text{z}}_{M}\right]}^{⊤}\in {\mathbb{R}}^{M\times N}$ and ${\text{V}}_{\text{OFM}}={\left[{\text{v}}_{1},\cdots \;,{\text{v}}_{M}\right]}^{⊤}\in {\mathbb{R}}^{M\times L}$ as follows:


$$\begin{array}{l} {\text{Z}}_{\text{OFM}}\\ =\left(\begin{array}{c} z_{1,1}^{\left(1\right)},z_{1,2}^{\left(1\right)},\cdots \;,z_{1,n}^{\left(1\right)},\;\cdots \;,z_{1,1}^{\left(l\right)},z_{1,2}^{\left(l\right)},\cdots \;,z_{1,n}^{\left(l\right)},\cdots \;,z_{1,1}^{\left(L\right)},z_{1,2}^{\left(L\right)},\cdots \;,z_{1,n}^{\left(L\right)}\\ \vdots \;\vdots \;\ddots \;\vdots \;\\ z_{i,1}^{\left(1\right)},z_{i,2}^{\left(1\right)},\cdots \;,z_{i,n}^{\left(1\right)},\;\cdots \;,z_{i,1}^{\left(l\right)},z_{i,2}^{\left(l\right)},\cdots \;,z_{i,n}^{\left(l\right)},\cdots \;,z_{i,1}^{\left(L\right)},z_{i,2}^{\left(L\right)},\cdots \;,z_{i,n}^{\left(L\right)}\\ \;\vdots \;\vdots \;\ddots \;\vdots \;\\ z_{M,1}^{\left(1\right)},z_{M,2}^{\left(1\right)},\cdots \;,z_{M,n}^{\left(1\right)},\;\cdots \;,z_{M,1}^{\left(l\right)},z_{M,2}^{\left(l\right)},\cdots \;,z_{M,n}^{\left(l\right)},\cdots \;,z_{M,1}^{\left(L\right)},z_{M,2}^{\left(L\right)},\cdots \;,z_{M,n}^{\left(L\right)}\\ \; \end{array}\right),\\ \text{and }{\text{V}}_{\text{OFM}}=\left(\begin{array}{c} v_{1,1},\;{\;v}_{1,2},\;\cdots \;,{\;v}_{1,L}\\ \vdots \;\vdots \;\ddots \;\vdots \;\\ v_{i,1},\;{\;v}_{i,2},\;\cdots \;,{\;v}_{i,L}\\ \vdots \;\vdots \;\ddots \;\vdots \;\\ v_{M,1},\;v_{M,2},\;\cdots \;,\;v_{M,L} \end{array}\right). \end{array}$$

**Figure 1:**
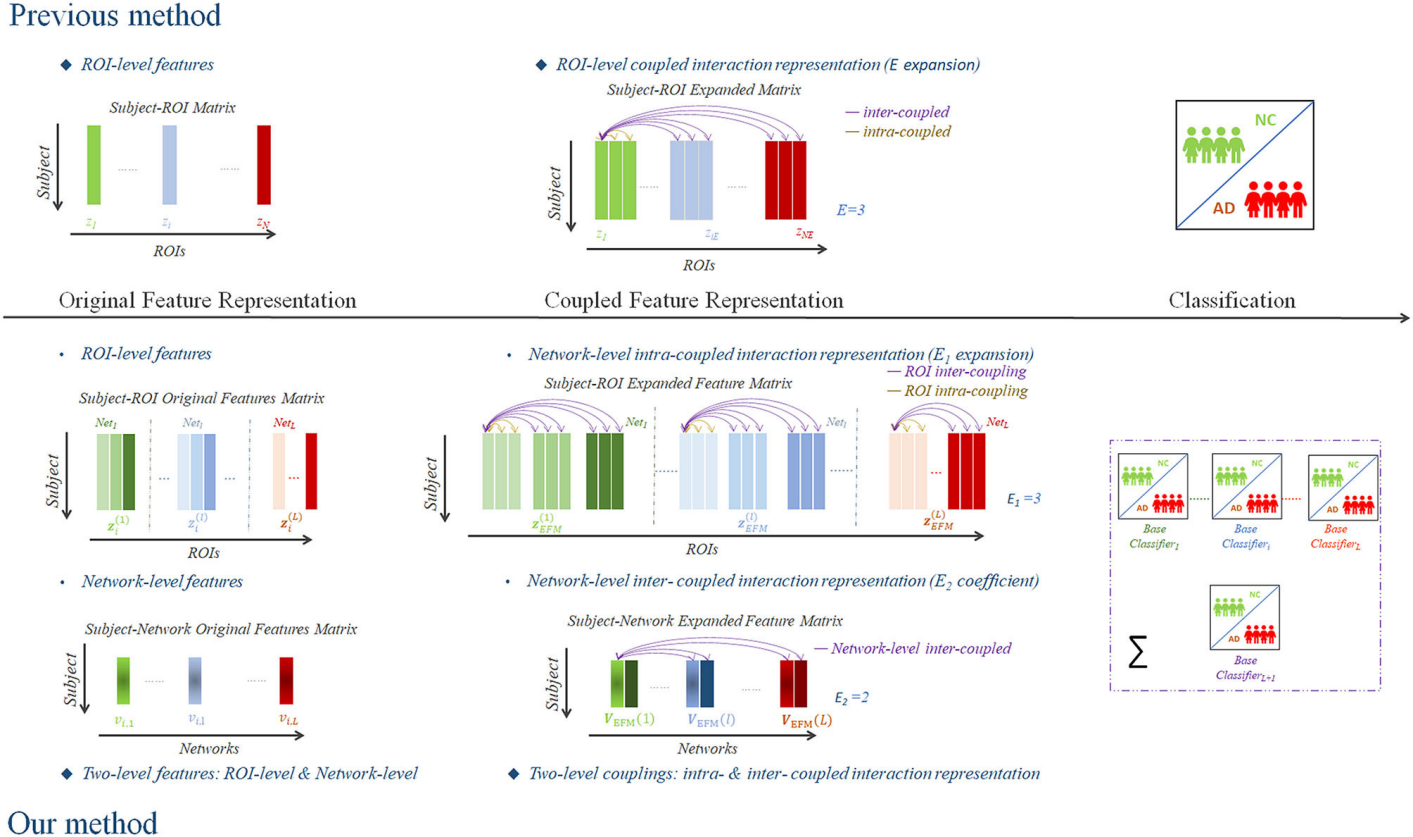
A scheme of the proposed framework of two-level coupled interaction representation of neuroimaging features.

Compared with the prior ROI-level coupled feature representation method (Shi et al., 2014), the present study proposed a novel two-level coupled feature representation method that attempted to investigate the complex coupling relationship of both the network-level feature matrix **V**_OFM_ and the ROI-level feature matrix **Z**_OFM_ for the identification of NC and AD using structural MRI data. We illustrated and schematized our framework in Figure 1 compared with the previous ROI-level coupled interaction representation method (Shi et al., 2014).

#### The Network-Level Intra-coupled Interactions

We illustrated the method of performing the network-level intra-coupled feature representation by the ROI-level feature matrix of the *l*_*th*_ brain network as an example.

Referring to previous study about the coupled attribute analysis on numerical data (Wang et al., 2013), the ROI-level feature vector of each brain network, ${\text{z}}_{i}^{\left(l\right)}$, was mapped into the expanded feature space, employing a matrix expansion with *E*_1_ power as follows:

$$\begin{array}{l} {}[{\langle z_{i,1}^{\left(l\right)}\rangle }^{1},\;{\langle z_{i,1}^{\left(l\right)}\rangle }^{2},\;\cdots {,\;\langle z_{i,1}^{\left(l\right)}\rangle }^{E_{1}},{\langle z_{i,2}^{\left(l\right)}\rangle }^{1},\;{\langle z_{i,2}^{\left(l\right)}\rangle }^{2},\\ \cdots {,\;\langle z_{i,2}^{\left(l\right)}\rangle }^{E_{1}},\cdots {\langle z_{i,n}^{\left(l\right)}\rangle }^{1}{,\langle z_{i,n}^{\left(l\right)}\rangle }^{2},\;\cdots {,\;\langle z_{i,n}^{\left(l\right)}\rangle }^{E_{1}}\;], \end{array}$$

and we can represent the ROI-level **E**xtended **F**eatures **M**atrix (EFM) of this brain network as follows:


$$\begin{array}{l} {\;\text{Z}}_{\text{EFM}}\in {\mathbb{R}}^{M\;\times \;\left(n\;\times {\;E}_{1}\right)}=\left(\begin{array}{c} {\langle z_{1,1}^{\left(l\right)}\rangle }^{1},\;{\langle z_{1,1}^{\left(l\right)}\rangle }^{2},\;\cdots {,\;\langle z_{1,1}^{\left(l\right)}\rangle }^{E_{1}},{\langle z_{1,2}^{\left(l\right)}\rangle }^{1},\;{\langle z_{1,2}^{\left(l\right)}\rangle }^{2},\;\cdots {,\;\langle z_{1,2}^{\left(l\right)}\rangle }^{E_{1}},\cdots {\langle z_{1,n}^{\left(l\right)}\rangle }^{1}{,\langle z_{1,n}^{\left(l\right)}\rangle }^{2},\;\cdots {,\;\langle z_{1,n}^{\left(l\right)}\rangle }^{E_{1}}\\ \vdots \;\vdots \;\ddots \;\vdots \;\\ {\langle z_{i,1}^{\left(l\right)}\rangle }^{1},\;{\langle z_{i,1}^{\left(l\right)}\rangle }^{2},\;\cdots {,\;\langle z_{i,1}^{\left(l\right)}\rangle }^{E_{1}},{\langle z_{i,2}^{\left(l\right)}\rangle }^{1},\;{\langle z_{i,2}^{\left(l\right)}\rangle }^{2},\;\cdots {,\;\langle z_{i,2}^{\left(l\right)}\rangle }^{E_{1}},\cdots {\langle z_{i,n}^{\left(l\right)}\rangle }^{1}{,\langle z_{i,n}^{\left(l\right)}\rangle }^{2},\;\cdots {,\;\langle z_{i,n}^{\left(l\right)}\rangle }^{E_{1}}\\ \vdots \;\vdots \;\ddots \;\vdots \;\\ {\langle z_{M,1}^{\left(l\right)}\rangle }^{1},\;{\langle z_{M,1}^{\left(l\right)}\rangle }^{2},\;\cdots {,\;\langle z_{M,1}^{\left(l\right)}\rangle }^{E_{1}},{\langle z_{M,2}^{\left(l\right)}\rangle }^{1},\;{\langle z_{M,2}^{\left(l\right)}\rangle }^{2},\;\cdots {,\;\langle z_{M,2}^{\left(l\right)}\rangle }^{E_{1}},\cdots {\langle z_{M,n}^{\left(l\right)}\rangle }^{1}{,\langle z_{M,n}^{\left(l\right)}\rangle }^{2},\;\cdots {,\;\langle z_{M,n}^{\left(l\right)}\rangle }^{E_{1}}\\ \; \end{array}\right). \end{array}$$

Next, the Pearson's correlation coefficient, *R*, between each pair of the ROI-level features of **Z**_EFM_, was calculated as the network-level intra-coupled weight matrix to reflect the ROI-level (intra- and inter-) coupled interactions within each brain network from both the linear and non-linear aspects. If the *p*-value of *R* was >0.05, the correlation coefficient was revised to 0. In this way, **R**^intra^ describes the correlation between the *k*_*th*_ ROI-level original feature and its own expanded powers, and **R**^inter^ describes the pairwise correlation between the *k*_*th*_ ROI-level feature and all the expanded powers of the others, as follows:

$$\begin{array}{l} {\text{R}}^{\text{intra}}\left(k\right)\in {\mathbb{R}}^{E_{1}\times E_{1}}=\left(\begin{array}{c} \gamma_{11}{,\gamma }_{12}\;,\cdots {,\gamma }_{1E_{1}}\\ \gamma_{21},{\;\gamma }_{22},\cdots {,\gamma }_{2E_{1}}\\ \vdots \;\ddots \;\vdots \;\\ \gamma_{E_{1}1\;},\;\gamma_{E_{1}2},\cdots \;,\gamma_{E_{1}E_{1}} \end{array}\right)\\ \text{and }{\text{R}}^{\text{inter}}\left(k\right)\in {\mathbb{R}}^{{\text{E}}_{1}\times \left({\text{E}}_{1}\times \left(\text{n}-1\right)\right)}=\left(\begin{array}{c} \cdots \;,\;\delta_{12}^{k,\tau },{\cdots \;,\delta }_{1E_{1}}^{k,\tau },\cdots \\ {\cdots \;\delta }_{21}^{k,\tau }{\;\cdots \;\delta }_{2E_{1}}^{k,\tau }\;\cdots \\ \vdots \;\vdots \;\vdots \;\\ {\cdots \;\delta }_{E_{1}1\;}^{k,\tau }{\cdots \;\delta }_{E_{1}E_{1}}^{k,\tau }\;\cdots \end{array}\right), \end{array}$$

where γ_*pq*_ is the revised Pearson's correlation coefficient between the *p*_*th*_ and *q*_*th*_ power of the *k*_*th*_ ROI-level original feature,${\;\langle z_{:,k}^{\left(l\right)}\rangle }^{p}$ and ${\langle z_{:,k}^{\left(l\right)}\rangle }^{q}$, respectively, and $\delta_{pq}^{k,\tau }$ is the revised Pearson's correlation coefficient between ${\langle z_{:,k}^{\left(l\right)}\rangle }^{p}$ and ${\langle z_{:,\tau }^{\left(l\right)}\rangle }^{q}$ (*k* ≠ τ).

For the *l*_*th*_ brain network from the *i*_*th*_ subject, the network-level intra-coupled feature vector can be represented as ${\text{u}}_{i}^{\left(l\right)}\in {\mathbb{R}}^{\left(n\times E_{1}\right)}$. The expanded vector of the *k*_*th*_ ROI-level feature is ${\text{z}}_{\text{EFM}}^{\text{intra}}\left(i\right)=\left[{\langle z_{i,k}^{\left(l\right)}\rangle }^{1},\;{\langle z_{i,k}^{\left(l\right)}\rangle }^{2},\;\cdots {,\;\langle z_{i,k}^{\left(l\right)}\rangle }^{E_{1}}\right]$, and the expanded vector of other features is

$$\begin{array}{l} {\text{z}}_{\text{EFM}}^{\text{inter}}\left(i\right)=[{\langle z_{i,1}^{\left(l\right)}\rangle }^{1},\;{\langle z_{i,1}^{\left(l\right)}\rangle }^{2},\;\cdots {,\langle z_{i,1}^{\left(l\right)}\rangle }^{E_{1}},\cdots {\langle z_{i,\tau }\rangle }^{1},\;{\langle z_{i,\tau }^{\left(l\right)}\rangle }^{E_{1}},\\ \cdots {,\langle z_{i,\tau }^{\left(l\right)}\rangle }^{E_{1}},\cdots {\langle z_{i,n}^{\left(l\right)}\rangle }^{1}{,\langle z_{i,n}^{\left(l\right)}\rangle }^{2},\;\cdots {,\;\langle z_{i,n}^{\left(l\right)}\rangle }^{E_{1}}],\;\left(k\neq \tau \right). \end{array}$$

Finally, the *k*_*th*_ ROI-level coupled feature vector of the *i*_*th*_ subject for the *l*_*th*_ brain network is denoted as follows:

$$\begin{array}{l} {\text{u}}_{i}^{\left(l\right)}\left(k\right)={\text{z}}_{\text{EFM}}^{\text{intra}}\left(i\right)⊙\omega ⊗{\left[R^{\text{intra}}\left(k\right)\right]}^{T}\\ +{\text{z}}_{\text{EFM}}^{\text{inter}}\left(i\right)⊙\overset{n-1}{\overset{︷}{\left[\omega ,\omega .\ldots ,\omega \right]}}⊗{\left[R^{\text{inter}}\left(k\right)\;\right]}^{T}, \end{array}$$

where $\omega =\;\left[\frac{1}{1!},\frac{\;1}{2!},\;\cdots \;,\frac{\;1}{E_{1}!}\;\right]$,

Then, the network-level intra-coupled feature matrix (CFM) of the *l*_*th*_ brain network for the *i*_*th*_ subject is:

$$\begin{array}{l} {\text{u}}_{i}^{\left(l\right)}=\left[{\text{u}}_{i}^{\left(l\right)}\left(1\right),{\text{u}}_{i}^{\left(l\right)}\left(2\right),\ldots ,{\text{u}}_{i}^{\left(l\right)}\left(n\right)\right]\in {\mathbb{R}}^{\left(n\times E_{1}\;\right)}. \end{array}$$

The network-level intra-coupled feature vector of the *i*_*th*_ subject is the concatenation of all networks' coupled features vectors, as follows:

$$\begin{array}{l} {\text{U}}_{i}=\left[{\text{u}}_{i}^{\left(1\right)},{\text{u}}_{i}^{\left(2\right)}\ldots \ldots ,{\text{u}}_{i}^{\left(L\right)}\right]\in {\mathbb{R}}^{\left(N\times E_{1}\right)}. \end{array}$$

Note that the interactions on ROIs belonging to different networks are not included.

#### The Network-Level Inter-Coupled Interactions

For the network-level inter-coupled representation, we can represent the CFM as **F** ∈ ℝ^*M* × *L*^ with the first *E*_2_ coefficients from CCA. In contrast to the network-level intra-coupled feature representation used with the revised Pearson's correlation coefficient, we chose the CCA coefficients as the coupling weights matrix for the network-level coupled feature representation. CCA is a way of inferring information from cross-covariance matrices of different network-level features. The canonical correlation for the canonical variate pairs from any two network-level ${{\text{Z}}_{\text{OFM}}}^{\left(l\right)}$ is as follows: ${\;\text{w}}_{l_{1}l_{2}}=R_{CCA}\left({{\text{Z}}_{\text{OFM}}}^{\left(l_{1}\right)},{{\text{Z}}_{\text{OFM}}}^{\left(l_{2}\right)}\right),\;\left(l_{1}\neq l_{2}\right)$, which can represent the inter-coupled interactions of different network-level features. The top *E*_2_ canonical correlations are used for inter-coupled interactions description rather than simply involving the whole **w**_*l*_, revised as ${\tilde{\text{w}}}_{l}={\tilde{R}}_{CCA}\left({{\text{Z}}_{\text{OFM}}}^{\left(l_{1}\right)},{{\text{Z}}_{\text{OFM}}}^{\left(l_{2}\right)}\right)$. For the whole brain, the network-level inter-coupled feature vector of the *i*_*th*_ subject is denoted as follows:

$$\begin{array}{l} {\text{f}}_{i}\left(w\right)={\text{V}}_{\text{EFM}}\left(W_{l}\right)⊙\overset{L-1}{\overset{︷}{\left[\omega ,\omega .\ldots ,\omega \right]}}⊗{\tilde{w}}_{l}, \end{array}$$

where $\omega =\;\left[\frac{1}{1!},\frac{\;1}{2!},\;\cdots \;,\frac{\;1}{E_{2}!}\right]$ and ${\text{f}}_{i}=\left[{\text{f}}_{i}\left(1\right),{\text{f}}_{i}\left(2\right),\cdots \;,{\text{f}}_{i}\left(L\right)\right]\in \;{\mathbb{R}}^{L}$.

To obtain the coupled feature representation at two levels, the final **CFM**_*network*_ for the *i*_*th*_ subject can be represented as follows: $\left[{{\text{U}}_{i},\;\text{f}}_{i}\right]\in {\mathbb{R}}^{\left(N\times E_{1}+L\;\right)}$.

### Classification With Coupled Features

Boosting is a machine learning approach based on the idea of improving the accuracy of a decision by combining many relatively weak base learners (Schapire, 2013). The AdaBoost algorithm works by updating parameters of feature distribution in weak learners over training samples after each iteration sequentially and adaptively (Freund and Schapire, 1997; Collins et al., 2002). In this study, the two-level coupled feature matrix was represented for the following classification analysis. We chose an SVM classifier with a linear kernel function as the base learner. In total, *L* + 1 base learners were trained, of which the *L* base learners were trained for different brain network-level intra-coupled features, and one was trained for the brain network-level inter-coupled matrix.

We carried out separate analyses on two tasks: AD vs. NC and MCI-c vs. MCI-nc classification. First, the boosting models were constructed on the two-level **C**oupled **F**eatures **M**atrix, denoted as **CFM**_network_ for AD vs. NC, and MCI-c vs. MCI-nc classification. The 10-fold cross-validation was applied to evaluate the performance, and the average results were reported. In our two-level coupled feature representation and classification scheme, several parameters need to be set, including *E*_1_ for the parameter of network-level intra-coupled expansion and *E*_2_ for network-level inter-coupled coefficient selection. Here, the optimal values of *E*_1_ and *E*_2_ were searched from a small set of {2, 3, 4} and {1, 2, 3, 4, 5}, respectively.

We also constructed three other kinds of feature matrices separately: (1) The ROI-level **O**riginal **F**eatures **M**atrix only, denoted as OFM_ROI_; (2) the ROI-level **C**oupled **F**eatures **M**atrix across the whole brain without the network-level information, denoted as **CFM**_ROI_; and 3) the network-level **O**riginal **F**eatures **M**atrix without coupling interaction information, denoted as **OFM**_network_. To validate the advantage of the two-level coupled feature representation, we compared the classification performances with these three different brain features.

## Results

The number of estimated ICs was 49 for the AD and NC groups with the structural MRI data, and 21 ICs showed significant between-group differences with Bonferroni correction. The results of our two-level coupled feature representation and the classification model showed that the best prediction accuracy is 90.44%, sensitivity is 88.5%, and specificity is 93.67% for AD and NC groups and the best prediction accuracy is 87.72%, sensitivity is 84.16%, and specificity is 91.64% for the MCI-c and MCI-nc groups.

Based on the two-level coupled feature representation, Tables 2, 3 show the classification results and give the detailed results of the best parameters of *E*_1_ and *E*_2_ as references for future studies. When *E*_1_ and *E*_2_ were set as 3 and 2, the two-level coupled feature representation achieved the best performance for AD vs. NC classification. The same parameter selection is applicable to the MCI-c vs. MCI-nc distinction.

**Table 2:** Classification results for AD vs. NC with different kinds of feature representations.

**Feature representation**	**OFM_**ROI**_**	**OFM_**network**_**	**CFM**_**ROI**_								**CFM**_**network**_									
**Parameters setting**			**E**_**1**_ **= 2**	**E**_**1**_ **= 3**	**E**_**1**_ **= 4**	**E**_**1**_ **= 2**					**E**_**1**_ **= 3**					**E**_**1**_ **= 4**				
						**E**_**2**_ **= 1**	**E**_**2**_ **= 2**	**E**_**2**_ **= 3**	**E**_**2**_ **= 4**	**E**_**2**_ **= 5**	**E**_**2**_ **= 1**	**E**_**2**_ **= 2**	**E**_**2**_ **= 3**	**E**_**2**_ **= 4**	**E**_**2**_ **= 5**	**E**_**2**_ **= 1**	**E**_**2**_ **= 2**	**E**_**2**_ **= 3**	**E**_**2**_ **= 4**	**E**_**2**_ **= 5**
ACC (%)	69.21	71.29	**73.51**	71.77	68.27	71.2	76.84	81.72	76.09	70.85	85.02	**90.44**	83.3	77.94	72.47	77.47	87.32	81.13	75.19	70.2
SEN (%)	67.29	67.72	**69.32**	69.57	64.03	68.67	74.12	76.75	72.41	68.29	82.28	**88.5**	81.08	74.04	68.01	79.4	85.72	77.42	71.01	67.18
SPE (%)	71.60	73.20	**75.42**	74.02	70.57	73.9	79.61	83.57	80.62	73.16	87.75	**93.67**	86.1	80.07	74.51	81.13	90.04	84.91	77.38	72.55

*OFM_ROI_, the original ROI-level feature representation; OFM_network_, the original network-level feature representation; CFM_ROI_, the coupled ROI-level feature representation; CFM_network_, the two-level feature representation; Acc, accuracy; Sen, sensitivity; Spe, specificity. The values with the highest accuracy are highlighted in boldface. The shadow of gray color is used to visually differentiate columns of the table*.

**Table 3:** Classification results for MCI-c vs. MCI-nc with different kinds of feature representations.

**Feature representation**	**OFM_**ROI**_**	**OFM_**network**_**	**CFM**_**ROI**_								**CFM**_**network**_									
**Parameters setting**			**E**_**1**_ **= 2**	**E**_**1**_ **= 3**	**E**_**1**_ **= 4**	**E**_**1**_ **= 2**					**E**_**1**_ **= 3**					**E**_**1**_ **= 4**				
						**E**_**2**_ **= 1**	**E**_**2**_ **= 2**	**E**_**2**_ **= 3**	**E**_**2**_ **= 4**	**E**_**2**_ **= 5**	**E**_**2**_ **= 1**	**E**_**2**_ **= 2**	**E**_**2**_ **= 3**	**E**_**2**_ **= 4**	**E**_**2**_ **= 5**	**E**_**2**_ **= 1**	**E**_**2**_ **= 2**	**E**_**2**_ **= 3**	**E**_**2**_ **= 4**	**E**_**2**_ **= 5**
ACC (%)	64.15	68.62	**75.10**	73.81	71.31	70.24	72.38	78.92	76.07	73.25	80.75	**87.72**	82.31	74.59	71.76	71.22	78.63	79.41	75.71	70.65
SEN (%)	62.30	66.44	**72.23**	70.54	67.90	66.21	69.70	75.92	73.82	70.61	78.42	**84.16**	78.73	72.16	69.02	68.78	76.23	77.20	71.02	68.99
SPE (%)	66.74	71.46	**79.42**	76.42	73.54	72.44	76.07	81.13	79.16	77.25	84.57	**91.64**	84.79	79.85	75.59	75.28	81.78	82.18	78.29	73.06

*OFM_ROI_, the original ROI-level feature representation; OFM_network_, the original network-level feature representation; CFM_ROI_, the coupled ROI-level feature representation; CFM_network_, the two-level feature representation; Acc, accuracy; Sen, sensitivity; Spe, specificity. The values with the highest accuracy are highlighted in boldface. The shadow of gray color is used to visually differentiate columns of the table*.

The results of the comparison for four different brain feature representations for AD and NC classification are shown in Table 2. The best classification accuracies for different features are 69.21% for OFM_ROI_, 73.51% for CFM_ROI_, 71.29% for OFM_network_, and 90.44% for CFM_network_. Table 3 shows the results of the classification performances for the four kinds of feature matrices for the MCI-c and MCI-nc classification. The best classification accuracies for different features are 64.15% for OFM_ROI_, 75.10% for CFM_ROI_, 68.62% for OFM_network_, and 87.72% for CFM_network_.

## Discussion

The current study proposed a novel network-level inter-coupled representation approach, integrated the intrinsic coupled relationships of both the ROI-level and the brain network-level features, and then applied them to the classification of subjects with AD, MCI-c, and MCI-nc from the normal elderly individuals based on structural MRI data. By integrating the intra- and inter-coupled interactions among the ROI-level and network-level features, we obtained the innovative coupled neuroimaging features, **CFM**_network_ and achieved the optimal classification accuracy for both AD vs. NC and MCI-nc vs. MCI-c classification compared with the OFM_ROI_, **CFM**_ROI_, and **OFM**_network_. These results indicated the effectiveness of the coupled interaction representation among different levels of neuroimaging features. Furthermore, the best-coupled expansion parameter *E*_1_ was 3 for the network-level intra-coupled interaction, and the best-coupled coefficient selection *E*_2_ was 2 for the network-level inter-coupled interaction.

### Two-Level Coupled Feature Representation for AD and NC Classification

In the current study, we explored the coupled interaction representation of two-level (the ROI -level and the network- level) neuroimaging features on structural MRI data. For AD and NC classification, the **OFM**_network_ representation obtained slightly better performance (accuracy = 71.29%) than the OFM_ROI_ representation (accuracy = 69.21%), and the **CFM**_network_ representation achieved much greater accuracy (accuracy = 90.44%) than the **CFM**_ROI_ representation (accuracy = 73.51%). Overall, the network-level feature representations showed preferable results to the ROI-level features, which suggested the advantages of the network-level features in the AD classification task. A number of studies built classification models to distinguish patients with AD from NCs based on the single- level features from brain neuroimaging data, such as the ROI-level features (Zhang et al., 2011; Zhan et al., 2015; Rondina et al., 2018) or the network-level features (Yang et al., 2011). For example, to identify the conversion from normal elderly cognition to AD, Zhan et al. defined 90 ROIs and computed the mean GMV as the ROI-level feature matrix and achieved an accuracy of 83.83% (Zhan et al., 2015). The ROI-level features computed by the ratio of increased GMV have also been extracted from structural MRI data, and they obtained a classification accuracy of 76.11% between AD and NC (Rondina et al., 2018). Different from them, Wang et al. considered the corresponding coefficients of ICs decomposed using the ICA algorithm as the network-level features and got 80.7% accuracy with the SVM classifier for the diagnosis of individuals with AD and HC (Yang et al., 2011). In this study, we not only extracted the ROI-level features but also obtained the network-level features and integrated them. Although the measurements or definitions of original features in our study were different from those in the prior studies, our study attempted to integrate hierarchical features from sMRI for the classification of AD and NC.

Compared with the original features (OFM_ROI_ and OFM_network_), the coupled features (**CFM**_ROI_ and **CFM**_network_) helped improve the classification results in this study. Among the four kinds of feature representations, the **CFM**_network_ obtained the best classification performance of AD and NC (accuracy = 90.44%), which demonstrated the strengths of the integration of multilevel (the ROI level and the network level) coupled interaction representation of hierarchical features. It has been demonstrated that there were strong couplings, including the relations that exist explicitly or implicitly between source and destination entities, among values, attributes, and objects for numerical data (Wang et al., 2013; Cao, 2015). Wang et al. introduced the framework to quantify and integrate the intra-coupled and inter-coupled interactions with the original information from numerical data (Wang et al., 2013). Many studies indicated that the original neuroimaging features exhibited complex regional dependencies, and the features in different brain networks changed diversely along with the progression of MCI and AD (Liu et al., 2017c; Zheng et al., 2019; Lee et al., 2020). Inspired by these pieces of research, we quantitatively measured the network-level intra-coupled relationships and proposed the network-level inter-coupled interaction feature representation. Recently, several studies focusing on the coupled interactions for ROI-level features have been reported, in which they analyzed the ROI-level coupled relationships and appealed to the coupling analysis for numerical data (Shi et al., 2014, 2020). By hypothesizing that the ROI-level features (the average GMV) were related to each other in some ways, Shi et al. introduced the coupled interaction representation for the ROI-level features and adopted the coupled boosting algorithm to analyze the pairwise coupled-diversity correlation between modalities with the best performance of 86.% for AD and NC classification (Shi et al., 2014). Our model achieved higher accuracy of 90.44%, which illustrated the advantages of our two-level coupled interactions representation.

### Two-Level Coupled Interaction Representation for MCI-C and MCI-Nc Classification

MCI is an intermediate stage in the trajectory from normal cognition to AD and is important for the early diagnosis of AD (Ahmed et al., 2017; Arbabshirani et al., 2017; Thung et al., 2018). To classify MCI-c and MCI-nc, we integrated the intra-coupled and inter-coupled interactions among the ROI-level and network-level features with the best accuracy of 87.72% compared with other feature representations. Considering the GM density from structural MRI data as the voxel-level features, Wang et al. obtained an accuracy of 69.77% for MCI-c vs. MCI-nc based on informed Partial Least Square models (Wang et al., 2016). Based on 38 subcortical volumes as ROI-level features, Aleksandra et al. classified MCI vs. NC with the Random Forest model (Lebedeva et al., 2017). Apart from the slight differences in classifiers, a common practice in former studies was the straight concatenation of all ROI-level features as independent variables into the input feature matrix. However, these schemes lost sight of the complicated dependencies among ROI-level features (Guo et al., 2015) and the diversified and heterogenous changes for different structural networks (Sui et al., 2014; Liu et al., 2017b). Compared with the abovementioned studies, we believe that the proposed two-level coupled interaction integration method which was validated could be more powerful for the diagnosis of MCI conversion to AD with promising results.

### Methodological Considerations

ICA is a popular data-driven method to study brain functional networks (Damoiseaux et al., 2012) and structural networks (Guo et al., 2015; Liu et al., 2017c). The network-level features extracted by ICA could effectively reduce the data dimensions and depend entirely on brain neuroimaging data themselves without prior knowledge. It has been confirmed that ROIs in the same brain network carried similar and interregional covariate information and exhibited more complicated regional dependencies than those in different brain networks (Liu et al., 2017a; Filippi et al., 2020; Wang et al., 2022). Thus, we performed ICA to identify brain structural networks from AD and NC groups and defined the representation of the network-level and ROI-level neuroimaging features.

Then, we designed the two-level coupled interaction integration of hierarchical features to evaluate the network-level intra-coupled and inter-coupled effects in AD and MCI classification. More specifically, we innovatively considered both the network-level intra-coupled interaction for every network individually, quantified by the intra-coupled and inter-coupled interactions among the ROI-level features within this network but not ROIs across the whole brain; and the network-level inter-coupled interaction among different network-level features was captured by the coupled coefficients between the ROI-level feature set of this network and the ROI-level features set of others. Besides, CCA can maximize the correlation between a linear combination of the variables in two datasets and has been applied to identify the relationship between brain networks (Sui et al., 2012; Ouyang et al., 2015; Taquet et al., 2021). In this study, CCA was performed on the ROI-level feature sets of any two brain networks and obtained the inter-coupled coefficients of network-level features to avoid reusing the ROI-level features information for network-level coupled interaction representation.

In the current study, *E*_1_ was denoted as the maximal power for the expansion of the ROI-level features in the network-level intra-coupled interaction representation and *E*_2_ as the number of the CCA coefficients selected to express the information for the network-level inter-coupled interaction representation. In this way, we integrated the two-level coupled interactions, including both the intra-coupled and inter-coupled interactions for both the network-level and the ROI-level features. We set the range of *E*_1_, from 2 to 4, and *E*_2_, from 1 to 5, respectively. When the value of *E*_1_ increases, the value of *E*_1_! will grow correspondingly so will *E*_2_!. The coupled interactions for feature values are quantified by a Taylor-like expansion, $\omega =\;\left[\frac{1}{1!},\frac{\;1}{2!},\;\cdots \;,\frac{\;1}{E_{1}!}\right]$. Along with the increase of *E*_1_ and *E*_2_, the reciprocals, $\frac{\;1}{E_{1}!}$ and $\frac{\;1}{E_{2}!}$, decreased accordingly and caused the corresponding weight value of the expanded items to be too small to capture the interactions among different features. Furthermore, the greater *E*_1_ or *E*_2_ may have less significant effects on the classification performance. Then, the appropriate *E*_1_ or *E*_2_ helps to fully exploit the information of coupled interactions within hierarchical features. As our results indicated, the classification performance changed with the variation of the two coupled interaction parameters. When *E*_1_ = 3 and *E*_2_ = 2, the best result was obtained in this tudy, which implied that the information of coupled interactions within hierarchical features has been fully exploited. When *E*_1_ = 1, the number of the ROI-level features was still invariant, which meant that the ROI-level coupled feature matrix was the original ROI-level feature matrix without coupled interaction analysis. When *E*_1_ increased, the number of ROI features increased with *E*_1_-fold accordingly. When *E*_1_ was equal to 3, each ROI-level feature was expanded three times in numerical space than the original feature. The inter-coupled interaction parameter for brain network-level features indicated that the first *E*_2_ pairs of canonical variables *via* CCA were maximally adequate to express the information among brain network-level features. When *E*_2_ was equal to 2, the top two coefficients of CCA were selected for the network-level inter-coupled interaction representation. With regard to the ROI-level and network-level coupled interactions of parameters setting, we recommend *E*_1_ = 3 and *E*_2_ = 2 for similar analysis in the future.

### Limitations and Future Work

The current study focused on constructing a novel coupled relationship representation to combine the ROI-level and network-level features, and then, we only adopted the numerical features from the structural MRI data. As different neuroimaging modality features provide complementary information, the coupled interactions of different modalities are heterogeneous (Zhang et al., 2011; Rathore et al., 2017). The coupled interactions based on multi-modality features are a novel issue that needs more exploration. The representation and integration of the intra-coupled interaction and inter-coupled interaction at multilevels, including the modality level, the network level, and the ROI level, will be investigated in future studies.

## Conclusion

In the current study, we proposed a network-level inter-coupled interaction representation approach with the independent components from ICA as the network-level features and the CCA weights for network-level inter-coupled characteristics. Then, we integrated the ROI-level and network-level coupled interactions based on structural MRI data to classify subjects with AD, MCI-c, MCI-nc, and NC. Our results demonstrated that the two-level coupled interaction feature representation outperformed the original feature representation and the single-level coupled representation and provided a perspective based on the coupled interaction integration of hierarchical neuroimaging features.

## Data Availability Statement

Publicly available datasets were analyzed in this study. This data can be found at: adni.loni.usc.edu.

## Ethics Statement

The ADNI study was approved by the Institutional Review Boards/Research Ethics Boards of each participating site. All participants provided their written informed consent in this study. The ethics committees/institutional review boards providing the approval for the ADNI study are: Albany Medical Center Committee on Research Involving Human Subjects Institutional Review Board, Boston University Medical Campus and Boston Medical Center Institutional Review Board, Butler Hospital Institutional Review Board, Cleveland Clinic Institutional Review Board, Columbia University Medical Center Institutional Review Board, Duke University Health System Institutional Review Board, Emory Institutional Review Board, Georgetown University Institutional Review Board, Health Sciences Institutional Review Board, Houston Methodist Institutional Review Board, Howard University Office of Regulatory Research Compliance, Icahn School of Medicine at Mount Sinai Program for the Protection of Human Subjects, Indiana University Institutional Review Board, Institutional Review Board of Baylor College of Medicine, Jewish General Hospital Research Ethics Board, Johns Hopkins Medicine Institutional Review Board, Lifespan – Rhode Island Hospital Institutional Review Board, Mayo Clinic Institutional Review Board, Mount Sinai Medical Center Institutional Review Board, Nathan Kline Institute for Psychiatric Research & Rockland Psychiatric Center Institutional Review Board, New York University Langone Medical Center School of Medicine Institutional Review Board, Northwestern University Institutional Review Board, Oregon Health and Science University Institutional Review Board, Partners Human Research Committee Research Ethics, Board Sunnybrook Health Sciences Centre, Roper St. Francis Healthcare Institutional Review Board, Rush University Medical Center Institutional Review Board, St. Joseph's Phoenix Institutional Review Board, Stanford Institutional Review Board, The Ohio State University Institutional Review Board, University Hospitals Cleveland Medical Center Institutional Review Board, University of Alabama Office of the IRB, University of British Columbia Research Ethics Board, University of California Davis Institutional Review Board Administration, University of California Los Angeles Office of the Human Research Protection Program, University of California San Diego Human Research Protections Program, University of California San Francisco Human Research Protection Program, University of Iowa Institutional Review Board, University of Kansas Medical Center Human Subjects Committee, University of Kentucky Medical Institutional Review Board, University of Michigan Medical School Institutional Review Board, University of Pennsylvania Institutional Review Board, University of Pittsburgh Institutional Review Board, University of Rochester Research Subjects Review Board, University of South Florida Institutional Review Board, University of Southern, California Institutional Review Board, UT Southwestern Institution Review Board, VA Long Beach Healthcare System Institutional Review Board, Vanderbilt University Medical Center Institutional Review Board, Wake Forest School of Medicine Institutional Review Board, Washington University School of Medicine Institutional Review Board, Western Institutional Review Board, Western University Health Sciences Research Ethics Board, and Yale University Institutional Review Board.

## Author Contributions

KL, QL, LY, and XG conceived, designed the experiments, and drafted the manuscript. KL and QL analyzed the data. All authors contributed to the article and approved the submitted version.

## Funding

This work was supported by the National Natural Science Foundation of China (Grant Nos. 62071051 and 61671066) and the Key Program of the National Natural Science Foundation of China (Grant No. 61731003).

## Conflict of Interest

The authors declare that the research was conducted in the absence of any commercial or financial relationships that could be construed as a potential conflict of interest.

## Publisher's Note

All claims expressed in this article are solely those of the authors and do not necessarily represent those of their affiliated organizations, or those of the publisher, the editors and the reviewers. Any product that may be evaluated in this article, or claim that may be made by its manufacturer, is not guaranteed or endorsed by the publisher.
